# Metabolomics combined with transcriptomics analyses of mechanism regulating testa pigmentation in peanut

**DOI:** 10.3389/fpls.2022.1065049

**Published:** 2022-12-16

**Authors:** Xin Wang, Yue Liu, Lei Ouyang, Ruonan Yao, Dongli He, Zhongkui Han, Weitao Li, Yingbin Ding, Zhihui Wang, Yanping Kang, Liying Yan, Yuning Chen, Dongxin Huai, Huifang Jiang, Yong Lei, Boshou Liao

**Affiliations:** ^1^ Key Laboratory of Biology and Genetic Improvement of Oil Crops, Ministry of Agriculture and Rural Affairs, Oil Crops Research Institute of the Chinese Academy of Agricultural Sciences, Wuhan, China; ^2^ State Key Laboratory of Biocatalysis and Enzyme Engineering, School of Life Sciences, Hubei University, Wuhan, China

**Keywords:** peanut, seed coat, flavonoid, procyanadin, anthocyanin, metabolomics, phenylpropanoid

## Abstract

Peanut testa (seed coat) contains large amounts of flavonoids that significantly influence seed color, taste, and nutritional qualities. There are various colors of peanut testa, however, their precise flavonoid components and regulatory mechanism of pigmentation remain unclear. In this study, a total of 133 flavonoids were identified and absolutely quantified in the seed coat of four peanut cultivars with different testa color using a widely targeted metabolomic approach. Black peanut skin had more types and substantial higher levels of cyanidin-based anthocyanins, which possibly contribute to its testa coloration. Procyanidins and flavan-3-ols were the major co-pigmented flavonoids in the red, spot and black peanuts, while flavanols were the most abundant constitutes in white cultivar. Although the concentrations as well as composition characteristics varied, the content ratios of procyanidins to flavan-3-ols were similar in all samples except for white peanut. Furthermore, MYB-like transcription factors, anthocyanidin reductases (ANR), and UDP-glycosyltransferases (UGT) were found to be candidate genes involved in testa pigmentation *via* RNA-seq and weighted gene co-expression network analysis. It is proposed that UGTs and ANR compete for the substrate cyanidin and the prevalence of UGTs activities over ANR one will determine the color pattern of peanut testa. Our results provide a comprehensive report examining the absolute abundance of flavonoid profiles in peanut seed coat, and the finding are expected to be useful for further understanding of regulation mechanisms of seed coat pigmentation in peanut and other crops.

## 1 Introduction

Flavonoids represent a large class of polyphenolic compounds, and ubiquitously distributed in plant kingdom (Yonekura-Sakakibara et al., 2019). These metabolites contain a common 15-carbon skeleton (C6-C3-C6), and can be mainly divided into different subgroups, including flavonols, flavan-3-ols, anthocyanins, and proanthocyanidins (Alseekh et al., 2020). So far, more than 8000 flavonoids have been identified in plant species. The types and amounts of flavonoid vary greatly due to the differences in plant genotypes, tissues, and developmental stages (Wang et al., 2018a; Wen et al., 2020). Anthocyanins constitute an important group of naturally occurring pigments, and are responsible for the vivid colors of flowers, fruits and seeds in many species (Khoo et al., 2017). However, anthocyanins are highly unstable and susceptible to degradation due to several factors. Co-pigmentation with other flavones, flavonols, and their glycosides, stabilizes the structure of anthocyanins and consequently enhances their final coloration effects (Shi et al., 2021).

Peanut or groundnut (*Arachis hypogaea* L.) is a major oilseed and economic crop widely cultivated in many countries, and provides an important source of edible oil and protein for humans. The peanut kernel is surrounded by a paper-like substance known as testa (seed coat) or skin, which has been used in traditional Chinese medicine to treat chronic hemorrhages and bronchitis (Dean, 2020). It has been reported that peanut skins contain large amounts of polyphenolic compounds, including flavonoids and phenolic acids (de Camargo et al., 2017). Although the skin portions represent only 3-7% of the total weight of kernel, they were found to have 60-120 times higher total flavonoid contents (TFCs) compared to their cotyledon parts, contributing to about 90% of the TFCs in kernel depending on different varieties (Attree et al., 2015). There are various colors of peanut testa, including pink, red, purple, black, white, and multicolor, with pink and red being the most common. In recent years, black peanuts are more favored by consumers, because of their appealing color and additional health-beneficial ingredients in the skins. Anthocyanin extracts from black peanut testa show a board range of bioactivities due to their potent antioxidant properties (Peng et al., 2019). Previous studies have shown that the total anthocyanin contents (TACs) were considerable higher in deep-colored peanuts (black, purple) than those in light-colored (pink, red, white) cultivars, indicating TACs have a positive correlation with seed coat color (Chukwumah et al., 2009; Shem-Tov et al, 2012). Procyanidins are oligomers and polymers of flavan-3-ols, which exclusively consist of (-)-epicatechin and (+)-catechin. A-type procyanidins and their monomeric flavan-3-ols units were the major flavonoids present in peanut skins (Appeldoorn et al., 2009; Chukwumah et al., 2009; Sarnoski et al., 2012; de Camargo et al., 2017). However, the relationship between seed coat color and procyanidins contents in peanut remained inconclusive.

In previous studies, a non-targeted metabolomics approach was used to comparative analysis of the relative abundance of flavonoids among different peanut seed coat samples (Huang et al., 2019; Wan et al., 2020; Xue et al., 2021), however their precise components and absolute contents had less been studied. This has posed a major obstacle to the elucidation of the molecular basis of seed coat pigmentation. Recently, a modern widely targeted metabolomics method based on ultra-performance liquid chromatography-electrospray ionization-tandem mass spectrometry (UPLC-ESI-MS/MS) can systematically analyze metabolites, and has been extensively applied to biological studies (Wan et al., 2016; Li et al., 2020; Ai et al., 2021). In this study, we described identification and quantification of the major anthocyanins and their potential co-pigmented flavonoids in four peanut seed coats with different colors (red, white, black, and spot). Integrated metabolomics and transcriptomics were performed to identified key metabolites and/or genes regulating color formation in peanut skins. This work will certainly assist in efforts to improve the outward appearance and quality of peanut in future.

## 2 Materials and methods

### 2.1 Reagents and plant materials

HPLC-grade acetonitrile and methanol were purchased from Merck (Darmstadt, Germany). MilliQ water (Millipore, Bradford, USA) was used in all experiments. Formic acid and the standards were purchased from Sigma-Aldrich and MCE (MedChemExpress, USA). Four peanut cultivars with different testa color were used as materials in this study. “Zhonghua 9” (ZH9) is an elite peanut cultivar with black testa, and “Kangqibaihong” (KQBH) has white seed coat. The testa color of “HongHong” (HH) cultivar is red, while “Huapi” (HP) shows red with white spot in its seed coat. Peanut materials were planted in the test field of Oil Crop Research Institute (Wuchang), Chinese Academy of Agricultural Sciences, Wuhan, China. Seeds with similar size of the four cultivars were collected at different stages of development (from R4 to R8) (Boote, 1982). For the metabolomic and RNA-Seq (as well as qRT-PCR) experiments, testa was manually peeled from the seeds at the final mature stage (R8), immediately frozen in liquid nitrogen, and stored into -80°C refrigerator until use for analysis.

### 2.2 Total flavonoid and anthocyanin content measurement

Total flavonoid content in peanut seed coat was determined by the Plant Flavonoid Detection Kit from Beijing Solarbio Science & Technology Co., Ltd. (Beijing, China). Briefly, testa samples derived from R4 to R8 developmental stages were ground into powders in liquid nitrogen, then about 0.1 g powders were extracted overnight at 4°C with 1.0 ml 70% aqueous methanol. The mixtures were centrifuged at 12,000 rpm for 10 mins, and the absorbance of supernatants was measured at 470 nm. Flavonoid content was qualified by using a calibration curve of rutin with linearity range from 0.04 to 2.5 mg/mL (R2 > 0.99) as described in the manual protocol. Total anthocyanin content in peanut testa was assayed using Plant Anthocyanidin Determination Kit from Shanghai yuanye Bio-Technology Co., Ltd (Beijing, China) according to user’s manual. In brief, testa samples were ground into powders in liquid nitrogen, then about 0.1 g powders were mixed with 2 mL Anthocyanidin Assay Buffer (75% methanol, 25% water, and 0.5% acetic acid). The suspensions were vigorously vortexed for 5 min, sonicated for 30 min, and then placed at 4°C overnight. The mixtures were centrifuged at 12000 rpm for 10 mins. The absorbance of supernatants was read at 530 nm.

### 2.3 Widely targeted metabolomics analysis

The freeze-dried samples were crushed using a mixer mill MM 400 (Retsch, Germany) with a zirconia bead for 1.5 min at 30 Hz. 20 mg powders for each sample (with three biological replicates) were weighted and extracted with 0.5 mL 70% aqueous methanol. The extractions were sonicated for 30 min. After centrifugation at 12, 000 rpm under 4°C for 10 min, the supernatants were collected, and filtrated with a membrane (0.22 μm; Anpel) before UPLC-MS/MS analysis. An AB ExionLC™ Ultra-Performance Liquid Chromatography (UPLC) coupled with a QTRAP^®^ 6500+ MS System (AB, SCIEX, Framingham, MA, USA) was used for the quantification of target flavonoid metabolites. Separations were carried out using a Waters ACQUITY UPLC HSS T3 C18 column (100 mm×2.1 mm, pore size 1.8 µm) (Waters Corporation, Milford, USA). The mobile phase consisted of eluent A (water, 0.05% formic acid) and eluent B (acetonitrile, 0.05% acetic acid) at a flow rate of 0.35 mL/min with the temperature set at 40°C. The gradient elution program was set as follows: 0-1 min, 10%-20% B; 1-9 min, 20%-70% B; 9-12.5 min, 70%-95% B;12.5-13.5 min, 95% B; 13.5-13.6 min, 95%-10% B;13.6-15 min, 10% B. The injection volume is 2 μL. The effluent was analyzed by an ESI-triple quadrupole-linear ion trap-MS (ESI-Q TRAP-MS/MS). LIT (linear ion trap) and triple quadrupole scans in positive or negative ion mode were acquired on the LC/MS2 system, which was equipped with an ESI Turbo Ion-Spray interface. The optimized ESI parameters were as follows: ion source, turbo spray; temperature, 550°C; and ion spray (IS) voltage, 5500 V (Positive), -4500 V (Negative). Multiple reaction monitoring (MRM) experiments with the collision gas (nitrogen) of 35.0 kPa were performed to acquire QQQ scans. De-clustering potential (DP) and collision energy (CE) used for individual MRM transitions were assessed with further optimization.

### 2.4 Identification and quantitative analysis of flavonoid metabolites

Flavonoids were identified and annotated using a self-built database MWDB (MetWare biological science and Technology Co., Ltd. Wuhan, China) as well as the publicly available metabolite databases (Chen et al., 2013) according to their retention time (RT), MS2 spectrums obtaining from the MRM mode of QQQ mass spectrometry. The stock solutions of standards were prepared at the concentration of 5 μmol/L in 70% MeOH, and then diluted to a series of working solutions with gradient concentrations. The standard curves were established between the concentrations and their corresponding chromatographic peak areas obtaining from specific precursor ions in the MS2 spectra. The absolute contents of metabolites were calculated based on linear regression equations obtaining from standard curves (**Table S1**). Since a part of anthocyanin compounds were not commercially available so far, a calibration curve of delphinidin-3,5-*O-*diglucoside was used to calculate the concentrations of these anthocyanins. The experimental data were obtained from three independent biological replicates. For multivariate statistical analysis, Partial Least Squares-Discriminant Analysis (PLS-DA) was used to identified the alternation of metabolites among different groups. An absolute value of log2 (fold change) ≥ 1 and variable importance in the projection (VIP) > 1.0 were introduced to screen significant differential metabolites (SDMs).

### 2.5 Transcriptome sequencing and data analysis

Total RNA was extracted from peanut seed coats collected at the final mature stage (R8). RNA concentrations and purities were measured using NanoDrop 2000 (Thermo Fisher Scientific, Wilmington, DE). 1 μg of RNA per sample was used for preparing transcriptome library. The cDNA libraries were sequenced and constructed using the Illumina sequencing platform. Clean data (clean reads) were obtained by removing reads containing adapter, reads containing poly-N and low-quality reads from raw data. The clean paired-end reads were then mapped to the peanut reference genome (*Arachis hypogaea* cv. Tifrunner, version 2, https://www.peanutbase.org/peanut_genome) using HISAT2 software (version 2.0.5) (Kim et al., 2019). The multi-mapped reads were filtered out, and only uniquely mapped reads were used for quantification. The gene expression levels were estimated by FPKM values (fragments per kilobase of transcript per million fragments mapped). Differentially expressed genes (DEGs) were screened using the DESeq2 R package (1.20.0) with the criteria of |log2FC (fold change) | > 1 and an adjusted *P-value* < 0.01 (Love et al., 2014). KEGG enrichment analysis of DEGs was performed by KOBAS software (Mao et al., 2005).

### 2.6 Verification of RNA-seq data by real-time quantitative RT-PCR

Total RNA was isolated from peanut seed coats using an EASYspin plant RNA extraction kit (Aidlab Biotechlogies, Co., Ltd, Beijing, China) according to the manufacturer’s instructions. Genomic DNA was removed from RNA samples by use of DNase I, then about 1 μg RNA were reverse transcribed into first strand cDNA with MMLV reverse transcriptase kit (Thermofisher Scientific, USA). qRT-PCR reactions were analyzed on Bio-Rad CFX96 RT-PCR Detection system (Bio-Rad, Hercules, CA, USA) using Hieff qPCR SYBR Green Master Mix (YEASEN, Shanghai, China). The relative transcript levels were quantified by the 2^-ΔΔCt^ method (Livak and Schmittgen, 2001), using *A. hypogaea* Actin gene (accession number: Aradu.W2Y55) as an internal standard gene for normalization. qRT-PCR primers were listed in **Table S2**.

### 2.7 Weighted gene co-expression network analysis

A weighted gene co-expression network analysis (WGCNA) was performed using the WGCNA R package (v1.68) (Langfelder and Horvath, 2008) based on FPKM values of the filtered DEGs. Network construction and module identification were conducted using the topological overlap measure (TOM) with following parameters: “weighted network” = unsigned, “soft thresholding power” =15, “minimum module size” = 30, “minimum height for merging modules” = 0.25. Then, the calculated module eigengenes and Pearson’s correlation coefficient values were used to determine the association of modules with the major metabolite contents for the 12 samples. The number of edges in a node represented the hubness of the gene. The gene networks and top 20 hub genes within a module were visualized by Cytoscape software (Shannon et al., 2003).

## 3 Results

### 3.1 Phenotypic characterization of peanut seed coats at different developmental stages

**Figure 1:**
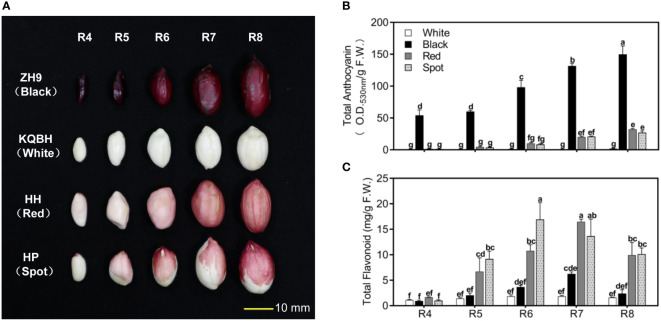
Phenotypic characterization of peanut seed coat at different pod developmental stages. **(A)** The morphological observation of peanut seed with different testa color (red, black, white and spot) from full pod stage (R4) to harvest maturity stage (R8). Analysis of total anthocyanin contents **(B)**, and total flavonoid contents **(C)** in peanut testa at different stages of development using spectrophotometric methods. Multiple comparisons in one-way ANOVA were done by Duncan grouping using SPSS Statistics tool (v22, IBM). Different letters marked in the figure indicate the difference among different samples as well as developmental stages. The bars correspond to standard errors of three biological replicates.

In general, peanut seed development can be divided into nine reproductive stages (R1-R9) (Boote, 1982). Four cultivar seeds with typical testa color were collected from full pod stage (R4) to harvest maturity stage (R8), respectively **(** **Figure 1A** **)**. Testa of ZH9 is black, and KQBH is white without pigment accumulation during seed maturation. The seed coat color of Honghong (HH) and Huapi (HP) are both light pink at the full pod stage (R4), and gradually get deeper during seed development. At the harvest maturity stage (R8), the seed coat of HH (Red) completely turns to red, while that of HP (Spot) is dark red with some white spots.

ZH9 with black testa had a large amount of total anthocyanin as early as the full pod stage (R4), whose contents were significantly higher than those of red (HH) and/or spot (HP) cultivars during the whole developmental stages **(** **Figure 1B** **)**. In contrast, a trace amount of anthocyanin was detected in white seed coat (KQBH), which is in accordance with its phenotype. Moreover, TFCs in the four cultivars increased from full pod stage (R4), and reached to their maximum levels at the stage of R6 or R7. At harvest maturity stage (R8), TFCs in red (HH) and/or spot (HP) peanut skin were much higher than those in black (ZH9) and/or white (KQBH) cultivars **(** **Figure 1C** **)**. These results suggest that peanut testa color is associated with anthocyanin as well as other flavonoid levels, which conforms with previous studies (Shem-Tov et al., 2012; Attree et al., 2015).

### 3.2 Metabolomics analysis of flavonoid profiling in peanut testa

#### 3.2.1 Comprehensive identification of flavonoids in peanut skins

**Figure 2:**
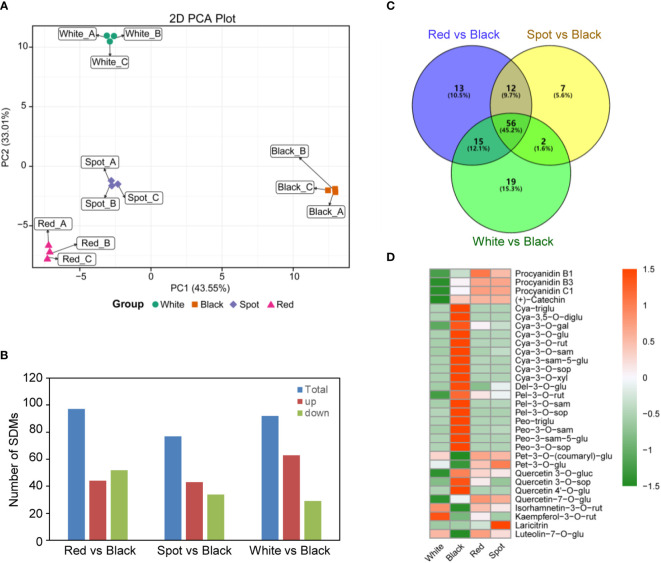
Metabolomics analysis of flavonoid profiles in peanut seed coats with different testa color based on UPLC-ESI-MS/MS. **(A)** Principal component analysis (PCA) of metabolomics data; **(B)** summary of significant differential flavonoid metabolites (SDMs) between black and other samples (Red *vs* Black, Spot *vs* Black, White *vs* Black); **(C)** venn diagram of SDMs, and **(D)** hierarchical clustering analysis of the abundance of 56 common SDMs shared by the three paired comparisons. The heatmap was drawn using the “pheatmap” package in the software R. Orange color indicates high abundance, while green is low.

To get more detailed information on the precise components of flavonoid involved in testa pigmentation, matured seed coats of the four peanut cultivars were collected at the harvest maturity stage (R8), and analyzed using UPLC-ESI-Q TRAP-MS/MS platform (Wang et al., 2018a; Li et al., 2020). In total, 133 flavonoid compounds were detected, including 47 anthocyanins, 21 flavonols, 21 flavones, 11 isoflavones, 9 flavanones, 7 flavanonols, 6 procyanidin, 6 flavan-3-ols and 5 chalcones (**Table S1**). To our knowledge, some of them were characterized for the first time in peanut skins, such as cyanidin-3-*O-*xyloside and delphinidin-3-*O-*(6’’-*O-*malonyl)-glucoside-3’-glucoside. Principal component analysis (PCA) showed a clear separation of flavonoid metabolites for peanut testa with different colors. Also, biological replicates for the same peanut cultivars grouped together, indicating a high reliability of the results (**Figure 2A**). Hierarchical clustering and correlation analysis displayed that the relationship between red (HH) and spot (HP) peanut was close, while black (ZH9) and white (KQBH) cultivar were quite distinct from the other samples (**Figure S1**). The significant differential metabolites (SDMs) were identified between different samples (**Table S3**). Compared with black peanut skins, the numbers of SDMs were shown in **Figure 2B**. There were 56 common SDMs shared by the three paired comparisons (Red *vs* Black, Spot *vs* Black, White *vs* Black), which mainly belong to anthocyanins, procyanidins, flavonols and their derivates (**Figures 2C, D**).

#### 3.2.2 Black peanut testa exhibited more types and higher levels of anthocyanin

**Figure 3:**
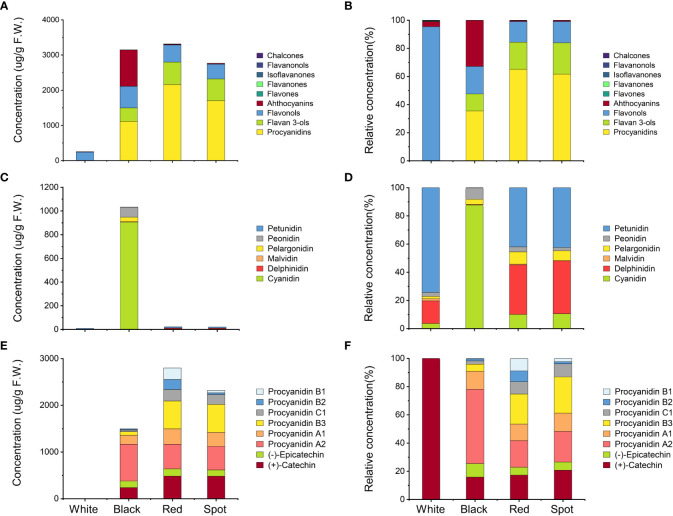
Absolute qualification and relative contents of different types of flavonoids in peanut seed coats. **(A)** Absolute, and **(B)** relative of concentrations of nine flavonoid subgroups; **(C)** absolute, and **(D)** relative of concentrations of six subclasses of anthocyanins. **(E)** absolute, and **(F)** relative of concentrations of oligomeric procyanidins and monomeric flavan-3-ols. The relative contents refer to the percentage of the amount of one component (or a subgroup of flavonoids) in the total amount of all components (or all subgroups of flavonoids) detected in the samples. The detail data were list in **Table S4**.

The 47 identified anthocyanins could be divided into six subgroups, including 12 cyanidin-, 11 delphinidin-, 10 peonidin-, 7 pelargonidin-, 5 petunidin- and 2 malvidin derivatives (**Table S1**). More types of anthocyanin compounds were found in black testa than the other samples. TAC in black peanut skins (ZH9) was as high as 1032.8 μg/g F.W., accounting for 32.82% of TFCs, while a small amount was measured in red, spot and white samples (**Figures 3A, B**; **Table S4**). Compared with other cultivars, there were substantially high levels of cyanidin- and peonidin based compounds in ZH9 (**Figures 3C, D**). According the fragmentation regularities of mass spectra and retention times, the most abundant anthocyanin compound was identified as cyanidin-3-*O-*sophoroside (529.7 ± 8.3 μg/g), accounting for over 50% of TACs in black peanut skin (**Table 1**; **Figure S2A**). On the other hand, in spite of low abundance, petunidin derivatives made up majority of TACs in white, red and spot peanut skins. For instance, petunidin-3-*O-*(coumaryl)-glucoside was found to be the richest anthocyanin in white peanut skins, but was not detected in black testa (**Table 1**). These results indicate that some specific anthocyanin compounds, such as cyanidin-3-*O-*sophoroside and petunidin-3-*O-*(coumaryl)-glucoside, could be used as metabolite biomarkers for identification of different peanut cultivars with distinct colors.

**Table 1:** The concentrations of major flavonoid compounds in different colored peanut skins.

Compound	White	Black	Spot	Red
**Procyanidin (Oligomer)**				
Procyanidin A1 (Type-A Dimer)	N.D.	191.4 ± 4.6	300.4 ± 7.2	328.6 ± 4.9
Procyanidin A2 (Type-A Dimer)	N.D.	787.9 ± 18.5	503.7 ± 21.9	527.7 ± 16.4
Procyanidin B1 (Type-B Dimer)	N.D.	5 ± 0.6	54.1 ± 3.4	245.9 ± 6.4
Procyanidin B2 (Type-B Dimer)	N.D.	20.3 ± 1.5	35.6 ± 2.3	216.6 ± 7
Procyanidin B3 (Type-B Dimer)	N.D.	76.2 ± 6.3	597.8 ± 8.8	595.9 ± 11.6
Procyanidin C1 (Type-B Trimer)	N.D.	35.2 ± 6.5	213.5 ± 18.6	244.4 ± 15.9
**Flavan-3-ol (Monomer)**				
(+)-catechin	0.09 ± 0.01	237.3 ± 4.4	482.8 ± 9.3	484.8 ± 7.7
(-)-Epicatechin	N.D.	142.9 ± 3.5	133.7 ± 1.3	155.5 ± 2.7
**Anthocyanin**				
Cyanidin-3-*O-*sophoroside	0.18 ± 0.01	529.7 ± 8.3	0.19 ± 0.05	0.31 ± 0.01
Cyanidin-3-*O-*sambubioside	0.12 ± 0.01	291.7 ± 4.0	0.97 ± 0.05	0.35 ± 0.01
Cyanidin-3-*O-*glucoside	N.D.	37.6 ± 1.0	0.12 ± 0.01	0.06 ± 0.01
Cyanidin-3,5-*O-*diglucoside	N.D.	10.1 ± 2.1	N.D.	N.D.
Cyanidin-3-*O-*sam-5-*O-*glucoside	N.D.	5.7 ± 0.5	0.03 ± 0.01	0.05 ± 0.01
Cyanidin-3-*O-*galactoside	N.D.	4.6 ± 0.2	0.69 ± 0.06	1.25 ± 0.01
Cyanidin-3-*O-*xyloside	N.D.	1.6 ± 0.1	0.01 ± 0.001	0.01 ± 0
Peo-3-*O-*sophoroside	0.06 ± 0.00	51.23 ± 1.76	N.D.	N.D.
Peo-3-*O-*sambubioside	N.D.	26.50 ± 1.10	0.22 ± 0.003	0.11 ± 0.00
Pet-3-*O*-(coumaryl)-glucoside	4.37 ± 0.20	N.D.	5.86 ± 0.29	6.70 ± 0.83
**Flavonol**				
Quercetin	0.02 ± 0.03	3.5 ± 0.3	4.3 ± 0.5	10.5 ± 0.7
Quercetin 3-*O-*glucuronide	2.6 ± 0.5	242.5 ± 13.5	51.7 ± 2.5	66.7 ± 1.1
Quercetin 3-*O-*sophoroside	0.4 ± 0	173.5 ± 5.1	0.67 ± 0.02	7.6 ± 0.2
Quercetin 3-*O-*galactoside	N.D.	58 ± 2.8	106.7 ± 1.9	137.5 ± 1.7
Quercetin-3-*O-*glucoside	13.1 ± 3.1	89 ± 4.1	94.6 ± 5.7	75.6 ± 4.3
Quercetin-3-*O-*rutinoside	42 ± 3.2	23.5 ± 0.3	72.2 ± 1.3	48.4 ± 0.4
Quercetin-3-*O-*arabinoside	N.D.	N.D.	1.1 ± 0.1	10.2 ± 0.4
Quercetin-7-*O-*glucoside	N.D.	1.3 ± 0	3 ± 0.1	3.8 ± 0.3
Kaempferol-3-*O-*rutinoside	13 ± 1.6	0.3 ± 0	0.67 ± 0.04	2.2 ± 0.04
Isorhamnetin	0.2 ± 0	0.8 ± 0.1	1.3 ± 0.2	5.9 ± 0.1
Isorhamnetin-3-*O-*rutinoside	166.1 ± 7.4	14.5 ± 0.4	77.2 ± 2.8	111.6 ± 4.1
Isorhamnetin 3-*O-*glucoside	2.2 ± 0.2	6.8 ± 0.1	4.9 ± 0.3	4.5 ± 0.2
**Flavone**				
Luteolin	0.04 ± 0.01	0.12 ± 0.04	0.21 ± 0.04	1.69 ± 0.12
**Flavanonol**				
Dihydroquercetin	0.05 ± 0.002	0.88 ± 0.02	1.21 ± 0.06	1.22 ± 0.03
**Flavanone**				
Eriodictyol	0.53 ± 0.08	0.39 ± 0.07	0.52 ± 0.06	2.94 ± 0.12
**Isoflavone**				
Prunetin	0.61 ± 0.06	0.15 ± 0.04	0.39 ± 0.05	1.16 ± 0.02

The concentration unit is μg/g fresh weight; N.D indicates not detected. Data were mean ± S.D. (standard deviation) derived from three biological replicates.

#### 3.2.3 Procyanidins and flavan-3-ols constituted majority of flavonoid contents in peanut skins

It was generally believed that peanut testa is a rich source of oligomeric procyanidins, including dimers, trimers and tetramers (Sarnoski et al., 2012). In this study, the most abundant flavonoid components were oligomeric procyanidins, which accounted for about more than half of TFCs in red (65.62%) and/or spot samples (61.65%) (**Figures 3E, F**). Procyanidins also made up 35.09% of TFCs in black testa, but was not detected in the white cultivar. To obtain deeper insights into procyanidin components in the extract of peanut skins, the fragment compositions and fragmentation regularities of mass spectra corresponding to individual parent ion were analyzed to confirmed their identifications. Based on the retention times and accurate of mass data measured by UPLC-ESI-MS, six procyanidin compounds were identified and qualified, including two type A and four type B oligomers (**Figures S2B–G**). All of them exhibited significantly higher amounts in the red and/or spot testa than those in black seed coats except for procyanidin A2 (**Table 1**). Procyanidin B3 was the most abundant procyanidin in the red (595.9 ± 11.6 μg/g) and spotted (597.8 ± 8.8 μg/g) peanut testa, while procyanidin A2 was shown to have the highest level in black skins (787.9 ± 18.5 μg/g). The content ratio of type-A procyanidins to type-B was 0.81 in red, and 1.17 in spot cultivar respectively, however, it reached up to 9.65 in black testa, which was nearly eight to ten-fold changes higher than those of red and spotted samples (**Figure S3**).

As basic building block for procyanidin oligomers, flavan-3-ols occupied the second rank of flavonoid component in red and spot testa, and accumulated in parallel to procyanidins (**Figures 3B, E, F**). There were six flavan-3-ols compounds measured in the samples, with (+)-catechin and (-)-epicatechin being the highest (**Figures S2H, J**). The amounts of (+)-catechin in red (484.8 ± 7.7 μg/g) and/or spot (482.8 ± 9.3 μg/g) peanut seed coats were about two folds more than that in black skins (237.3 ± 4.4 μg/g), while those of (-)-epicatechin in the three samples were more or less the same levels (from 133.7 ± 1.3 to 155.5 ± 2.7 μg/g) (**Table 1**). In contrast, there was no (-)-epicatechin in white peanut testa, but a trace amount of (+)-catechin was found. Hence, the sum contents of procyanidins and flavan-3-ols constituted majority of TFCs in spot (84.0%), red (84.4%) and black (47.5%) peanut skins except for white cultivar. These results suggest that procyanidin biosynthetic pathway was dominant in flavonoid metabolism and could be regulated coordinately in peanut testa.

#### 3.2.4 Flavonol metabolism was dominant in white peanut skins

Flavonols are the most widespread class of flavonoids in plants, and have been shown to possess remarkable bioactivities (Wen et al., 2020; Barreca et al., 2021). Apart from anthocyanins and proanthocyanins, flavonols are responsible for the testa color formation in *Arabidopsis* and bean (Beninger and Hosfield, 1998; Beninger and Hosfield, 1999; Pitura and Arntfield, 2019). Notably, flavonol was the most abundant flavonoid subgroup in white peanut skins (**Figures 3A, B**), and accounted for vast majority of TFCs (95.32%), with isorhamnetin-3-*O-*rutinoside (166.1 ± 7.4 μg/g) ranking the highest level (**Figure S2J** and **Table 1**). On the contrary, flavonol was the third abundant subgroup in black (19.56%), red (14.65%) and spot (15.17%) testa, with quercetin-based glycosides taking up the largest proportion. For instance, the richest flavonol in black skin was quercetin-3-*O-*glucuronide (242.5 ± 13.5 μg/g), and quercetin-3-*O-*galactoside (hyperoside) showed the highest levels in red and/or spot cultivars. These results suggested the accumulation and modification patterns of flavonol metabolites varied dramatically in different peanut cultivars, although they shared the common upstream biosynthetic pathway. The contents of remaining flavonoid subgroups, including flavones, flavanones, flavanonols and isoflavones, were relatively low (**Table S4**). Nevertheless, their levels were much higher in the red skins than those in other peanut cultivars. For instance, luteolin was the major component in flavone subgroup, its amount in red testa showed much higher than that in the other samples (**Table 1**). Similar results were also found for eriodictyol and prunetin.

### 3.3 Transcriptome analysis of peanut seed coats with different color

#### 3.3.1 Transcriptome sequencing and annotation

**Figure 4:**
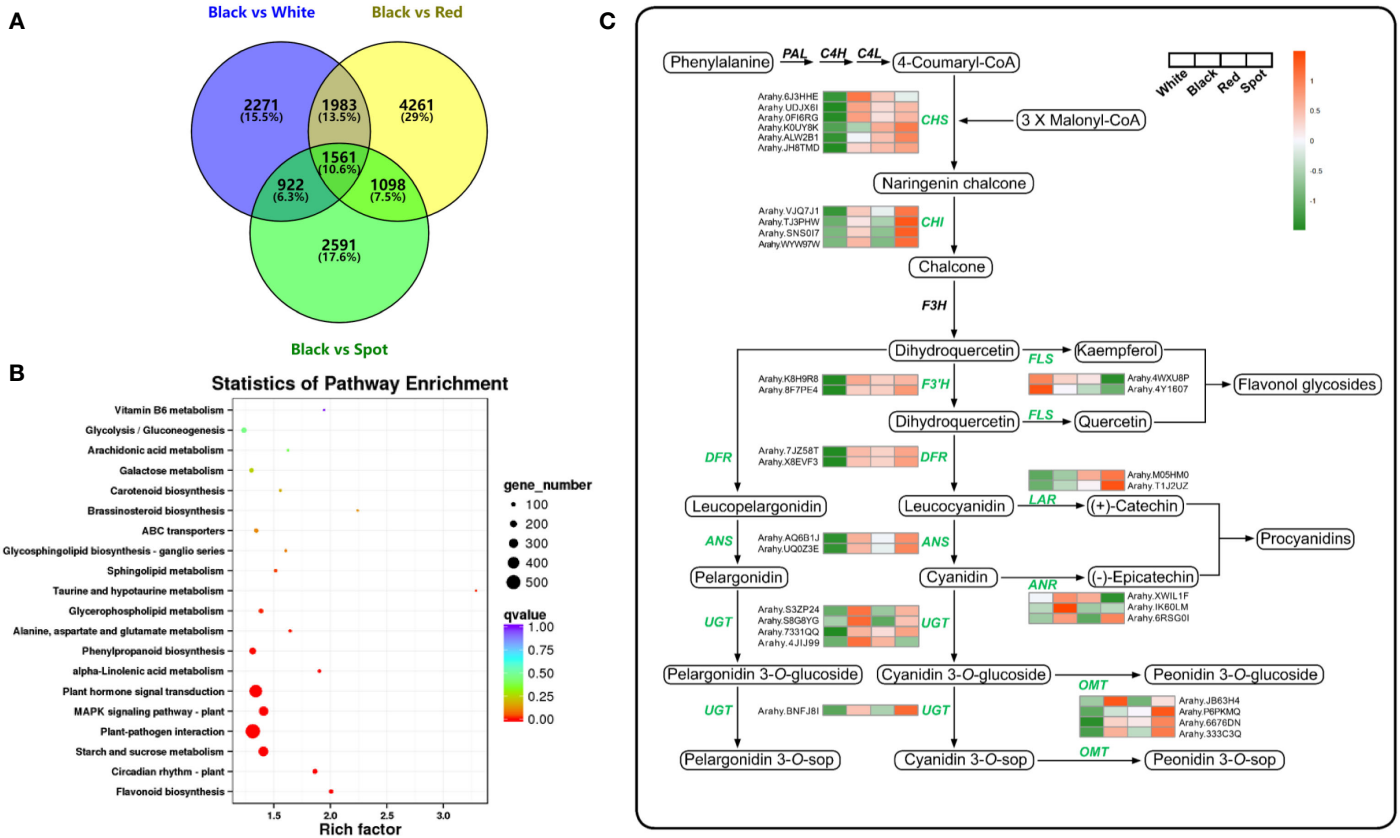
Transcriptome analysis of peanut seed coats with different color. **(A)** Venn diagram of differentially expressed genes (DEGs) between black peanut testa and other samples. **(B)** Pathway enrichment analysis of all DEGs. The dots represent the number of significant DEGs, the dots with different colors indicate different q-values. **(C)** Transcriptional expression pattern of structural enzyme genes involved in flavonoid biosynthetic pathway. The values in the heatmap represent the z-score of the FPKM values (transcript levels) in different samples. Orange color indicates high expression level, while green is low.

To study the molecular mechanisms underlying accumulation of anthocyanins and potential co-pigmented flavonoids in peanut skins, the total mRNA was subjected to construct RNA-seq library using Illumina paired-end platform. With three biological replicates per cultivars, the 12 samples yielded more than 101.5 Gb clean data with average Q30 score of 94.8% (**Table S5**), indicating highly reliable for further analysis. Differentially expressed genes (DEGs) were screened with the threshold of log2 |fold change| >1 and FDR < 0.05. There were 6737, 6172 and 8903 DEGs in the comparison sets: White *vs* Black, Spot *vs* Black and Red *vs* Black, respectively (**Figure S4**). As shown in the venn diagram, a total of 14688 DEGs were identified in at least one comparison set, and 1561 common DEGs could be detected in each set (**Figure 4A** and **Table S6**). All the DEGs were subsequently mapped to the KEGG database. “Flavonoid biosynthesis” (ko00941) was found to be the most enriched pathway, and “phenylpropanoid biosynthesis” (ko00940) involved in anthocyanin and flavonoid accumulation also ranked in the top ten pathway (**Figure 4B**).

#### 3.3.2 Analysis of structural genes involved in flavonoid biosynthesis

**Figure 5:**
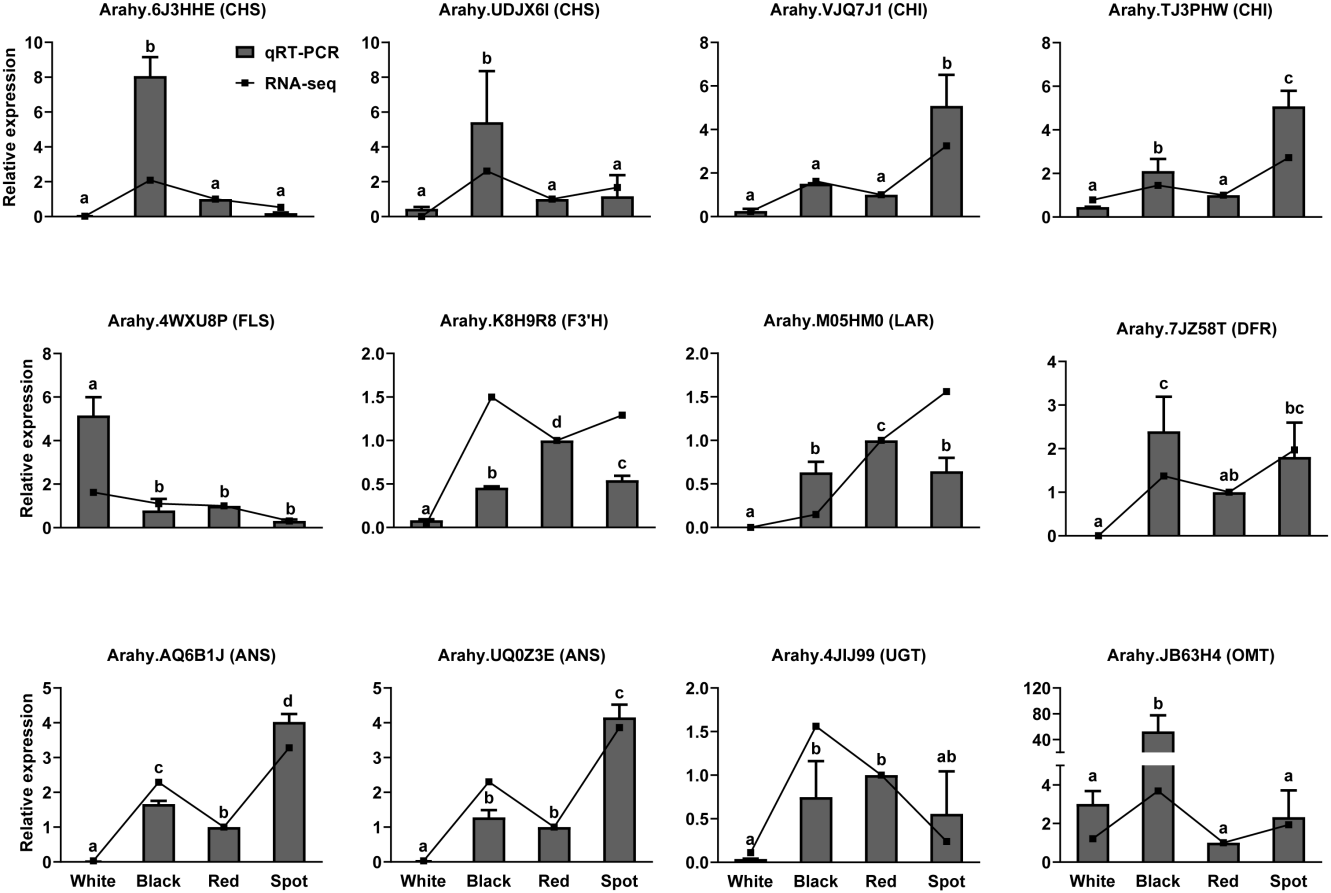
Quantitative RT-PCR (qRT-PCR) analysis of the expression levels of key enzyme genes involved in flavonoid biosynthetic pathway. The relative transcript levels among different samples were quantified by the 2^-ΔΔCt^ method, with *A. hypogaea* Actin gene as the reference gene for normalization. Multiple comparisons in one-way ANOVA were done by Duncan grouping using SPSS Statistics tool (v22, IBM). Different letters marked in the figure represent difference among different peanut cultivars. Error bars indicate the standard deviations of three biological replicates.

Flavonoids are synthesized *via* phenylpropanoid pathway under the control of multiple structural enzymes and regulatory genes, including chalcone synthase (CHS), chalcone isomerase (CHI), flavonol 3’-hydroxylase (F3’H), dihydroflavonol-4-reductase (DFR), and UDP-glycosyltransferase (UGT) (Dong and Lin, 2021). A total of 98 DEGs were assigned to “flavonoid biosynthesis” according to KEGG enrichment analysis results (**Table S7**). The expression pattern of flavonoid biosynthetic genes was examined in skins of the four representative cultivars (**Figure 4C**). CHS and DFR are the two committed enzymes in the early steps of flavonoid biosynthetic pathway. The transcript levels of five *AhCHS* and two *AhDFR* were much higher in black, red and spot cultivars than in white samples, which could probably drive more carbon flux from the upstream phenylpropanoids into flavonoid and/or anthocyanin metabolic processes. Consequently, TFCs were significantly higher in the three peanut samples (black, red and spot) than in white cultivars. On the other hand, flavonol synthase (FLS, arahy.4WXU8P, and arahy.4Y1607) was responsible for catalyzing the conversion of dihydroflavonols to flavonols. The significant higher level of FLS and lower of DFR in white peanut testa could redirect more carbon flux to the formation of flavonols, such as kaempferol and its glycosides. This probably explains why white skins had the highest proportion of flavonols among the four peanut varieties. Moreover, anthocyanidin synthase (ANS) and leucoanthocyanidin reductase (LAR) were both key enzymes involved in anthocyanidin and proanthocyanidin biosynthesis. They competed for the same substrate (leucocyanidin) to produce cyanidin and (+)-catechin, respectively. Compared with black peanut, the relative higher transcript abundance of LAR (Arahy.M05HM0) as well as lower expression level of ANS (Arahy.AQ6B1J) in the red seed coats would possibly contribute to more accumulation of (+)-catechin as well as procyanidin, which was in accordance with metabolomic data. In the tailing process, four UGTs and one *O-*methyltransferase (OMT) were found to have higher transcripts level in black peanut skins, which facilitate to generate more types of anthocyanidin derives and increase the stabilities of corresponding aglycones (Yang et al., 2018). Furthermore, the transcript levels of 12 structural genes were selected and analyzed by qRT-PCR, which had a good correlation with RNA-seq results and confirmed the reliability of transcriptome data (**Figure 5** and **Table S2**). As shown in the **Figure 5**, the transcript abundance of UGT (Arahy.4JIJ99) had no difference among the three samples (black, red and spot), while the levels of ANS (Arahy.UQ0Z3E and Arahy.AQ6B1J) were significant higher in spot than black peanut. Since the spot samples exhibited abundant (-)-epicatechin and procyanidins, it was proposed that the higher transcript abundance of ANS in spot samples will facilitate to generate cyanidin, some of which can be further converted into epicatechin by ANR, and finally be used for procyanidin biosynthesis.

#### 3.3.3 Identification of key WGCNA modules and genes related to flavonoid biosynthesis

**Figure 6:**
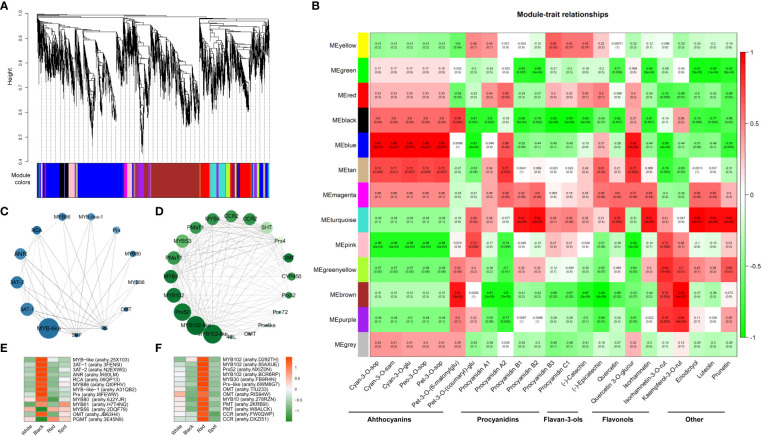
Identification of hub genes and key modules related to anthocyanin and other flavonoid metabolites in peanut seed coats by WGCNA. **(A)** Hierarchical cluster tree shows 13 modules of co-expressed genes. Different modules are marked with different colors. **(B)** Correlations of flavonoid contents with WGCNA modules. Each row corresponds to a specific module eigengene, and column to a trait. The values in each cell represent the correlation coefficients (r) and the *p-values* (in parentheses) of the module-trait association. The deeper the red or green in a cell, the higher the positive or negative correlation between the module eigengene and a trait feature. Visualization of co-expression network of blue **(C)** and turquoise **(D)** module by Cytoscape software. The top hub genes with the highest degree of connection to other genes are shown in nodes. The size of node corresponds to degree value, and color means module membership value calculated by WGCNA. The larger and darker of the nodes indicate the greater hubness of the gene. Hierarchical cluster analysis of the top hub genes in blue **(E)** and turquoise **(F)** module. The values in the heatmap **(E, F)** represent the z-score of the FPKM (transcript levels) in different samples. Orange color indicates high expression level, while green is low.

WGCNA is now commonly used to analyze transcriptome and metabolomics data for identifying trait-related co-expression modules (Langfelder and Horvath, 2008). To obtain a comprehensive understanding of the gene regulatory mechanism of flavonoid synthesis in peanut seed coats, WGCNA was conducted using FPKM values of the 14688 filtered DEGs to build a gene co-expression network (**Table S8**-**9**). Based on pairwise correlation analysis of gene expression level, 13 merged co-expression modules marked with different colors were shown in **Figure 6A**. Analysis of the module-trait relationships revealed that the major anthocyanin (eg. cyan-3-*O-*sop, cyan-3-*O-*sam) and flavonol (eg. quercetin-3-*O-*glucuronide) metabolites were tightly associated with “blue module” (R > 0.8 and *p-value* < 0.001), while two procyanidins (procyanidin B1 and B2) and three other flavonoids (eriodictyol, luteolin, and prunetin) were more related to “turquoise module” (**Figure 6B**). According to the values of WGCNA edge weight and node scores, the hub genes involved in regulating flavonoid biosynthesis were identified in the “blue” and “turquoise” module (**Figures 6C, D**). All the hub genes were shown in **Table S10**, which included several MYB transcript factors (eg. arahy.25X103, arahy.QI0PHV, arahy.6J2YJR, arahy.D292TH), and an anthocyanidin reductase (ANR, arahy.IK60LM). Most of hub genes in “blue module” were specifically up-regulated in black testa, while genes in “turquoise module” showed higher levels in red skins (**Figures 6E, F**). The expression profiles correlated well with the accumulation pattern of flavonoids in the four-peanut testa, these results further illustrated that the “blue module” was very relevant to anthocyanin synthesis, and “turquoise module” was related to procyanidin and other flavonoids metabolism.

## 4 Discussion

Flavonoids show a board range of bioactivities and health benefits due to their potent antioxidant properties (Levy et al., 2017; Chen et al., 2018; Yang et al., 2021). Peanut testa contains a large amount of flavonoid compound, which affects its nutritional and commercial value. Most of the flavonoids are colorless or pale yellow except for anthocyanins. To some extent, the content and composition of flavonoids also determine the color or pigmentation pattern of seed coats (Koes et al., 2005). Here, a total of 133 flavonoid metabolites were identified, and their absolute concentrations were measured based on the linear regression equations obtaining from corresponding standard curves. The results highlighted differential flavonoids produced by the four different cultivars, mainly in terms of metabolite types and abundances. We attempted to decipher the regulatory mechanisms of pigment formation in peanut through combining metabolomic approach with transcriptome analysis.

### 4.1 Content ratio of procyanidins to flavan-3-ols exhibits similar level in different peanut testa

Early investigations have shown type-A procyanidins predominated in peanut skins, while type-B were commonly found in important dietary sources such as apples and grapes (Zhang et al., 2013; Dudek et al., 2017; Rue et al., 2018). In current study, the amounts of type-A procyanidins were less than those of type-B in the red and spot cultivars, but type-A overwhelmed type-B in black peanut. These results indicated that the regulation mechanisms of procyanidin biosynthesis were distinct among different cultivars, and the differential accumulation pattern of procyanidins was possibly related to their color formation. Comparing the results for the different analyzed peanut skin samples (except for white testa), it is notable that the content ratio of procyanidin oligomers to their monomers (flavan-3-ols) displayed more or less the same levels although the absolute concentrations differed (**Table 1** and **Figure S3**). This aspect has also been observed in some other foods and nuts (De Pascual-Teresa et al, 2000; Rzeppa et al., 2011). Four beans belonging to the same genus in legumes, such as black bean and kidney bean, had different concentrations for the procyanidin and monomeric flavan-3-ol, as well as for the total amounts, but their relative compositions displayed a similar pattern (Bittner et al., 2013). The result suggests that the content ratio of procyanidins to monomeric flavan-3-ols might be characteristic for the different sample types and can be used for authenticity control, while their absolute concentrations varied possibly due to the origin of samples or stage of development.

### 4.2 MYB-like transcription factors and ANR play vital roles in seed coat pigmentation

In our transcriptome data, several MYB-like transcription factors (eg. MYB30, MYB80, MYB86, MYB102) and an ANR (arahy.IK60LM) were found to be hub genes involved in anthocyanin and proanthocyanin metabolism by WGCNA. Previous study showed that R2R3-MYB transcription factor and ANR were important regulatory genes that confer pigment accumulation in plant tissues (Yang et al., 2022). A major gene (*AhTc1*) controlling peanut purple testa was characterized to be encoding a R2R3-MYB transcription factor using QTL-seq method together with transcriptome analysis and gene expression profiling. Overexpression of *AhTc1* (arahy.J3K16L) in tobacco led to different degrees of purple color in leaves, flowers, fruits and testa (Zhao et al., 2020). Recently, a major location (*AhRt2*) controlling peanut testa color was fine-mapped to a 0.5 Mb genomic region on chromosome 12 using the BSA−seq and linkage mapping approaches, and the ANR (arahy.IK60LM) was suggested to be the possible candidate gene within this region (Zhang et al., 2022), which was in accordance with our WGCNA analysis.

### 4.3 Glycosylation of anthocyanidin *via* UGT contributes to testa coloration

In the tailing process, anthocyanidins are converted into colored pigments *via* glycosylation catalyzed by UGTs. Compared with other cultivars, black peanut had substantially higher level of anthocyanidin 3-*O-*glycoside, which is a main stable colored pigment in several plant tissues. Based on the transcriptome data, four putative UGTs and three ANRs were identified to be involved in testa pigmentation. The *UGT* transcripts have relative higher expression level in black peanut. It is proposed that UGTs and ANR compete for the substrate cyanidin (**Figure 4C**), and the prevalence of UGTs activities over ANR one will determine the color pattern of peanut testa. Moreover, cyanidin 3-*O-*sophoroside and cyanidin 3-*O-*sambubioside are the two major anthocyanins in black peanut skin, indicating glycosyl extension from anthocyanidin 3-*O-*glucosides plays a vital role in testa pigment formation (Zhao et al., 2017; Huang et al., 2019). Two specific UGTs (Ib3GGT and At3GGT) were characterized in Arabidopsis and purple sweet potato. The two enzymes were responsible for catalyzing the conversion of anthocyanidin 3-*O-*glucosides to anthocyanidin 3-*O-*sophorosides or anthocyanidin 3-*O-*sambubioside using UDP-glucose or UDP-xyloside as a sugar donor, respectively (Yonekura-Sakakibara et al., 2012; Wang et al., 2018b). Five peanut UGTs were found to have high sequence similarities with Ib3GGT and At3GGT, and putatively annotated as anthocyanidin 3-*O-*glucoside *2’’-O*-glycosyltransferase (**Figure S5A**). Phylogenetic analysis showed that AhUGT236 (arahy.BNFJ8I) exhibited the most closed relationship with Ib3GGT, and its transcript level was higher in black testa than red and white samples (**Figures S5B, C**). These results indicated AhUGT236 was possibly a candidate gene involved in the biosynthesis of anthocyanidin 3-*O-*sophoroside, and might play vital roles in testa pigmentation. However, the gene function and its regulatory mechanism warrant further investigation in future studies.

## 5 Conclusion

In this study, we integrated widely targeted metabolomics and transcriptomics analyses to elucidate the regulatory mechanism of flavonoid biosynthesis in testa of four peanut with different seed colors. The abundant accumulation of cyanidin-based anthocyanins contributes to coloration of black peanut testa. Procyanidins and flavonols were the major co-pigmented flavonoids present in peanut skins. Some specific anthocyanin compounds as well as the content ratio of procyanidins to monomeric flavan-3-ols could be used as metabolite biomarkers or authenticity control for identification of different peanut cultivars with distinct colors. These findings are expected to be useful for further understanding of regulation mechanisms of seed coat pigmentation in peanut and other crops.

## Data availability statement

The data presented in the study are deposited in the NCBI repository. accession number PRJNA885071.

## Author contributions

XW, YuL, LO, RY, ZH, WL and YD conducted the experiments. HJ, YoL and BL organized and supervised the overall project. DLH, LY, ZW, YK, DXH and YC performed the data analysis, XW wrote the manuscript. BL edited the manuscript. All authors contributed to the article and approved the submitted version.
