# Supplementary material for: Metabolomics combined with transcriptomics analyses of mechanism regulating testa pigmentation in peanut
Source: Front Plant Sci. 2022 Dec 16;13:1065049. doi: 10.3389/fpls.2022.1065049 (PMC9800836; doi:10.3389/fpls.2022.1065049)
Supplement: Supplementary file 11 [file DataSheet_1.pdf]

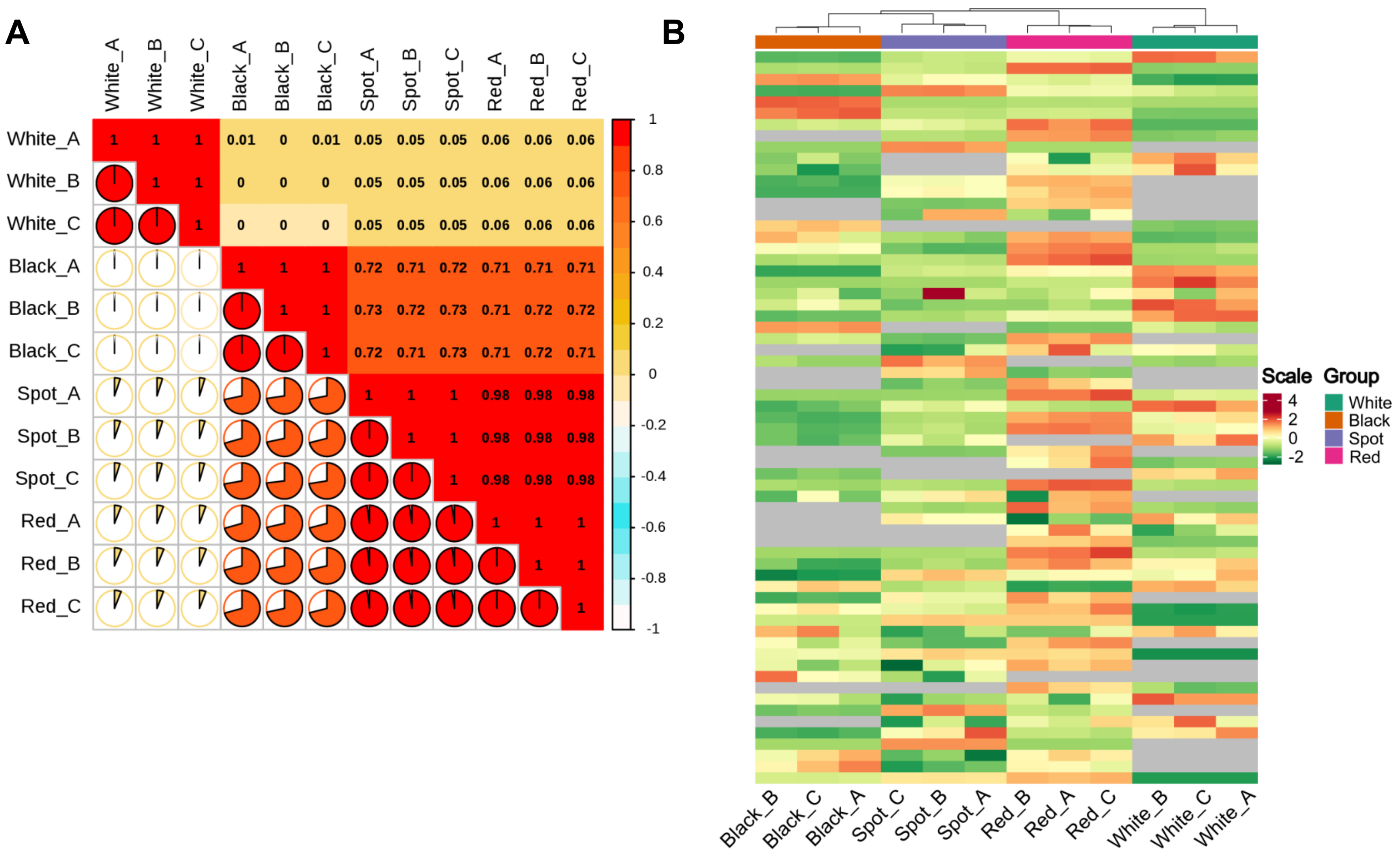

Figure S1. Correlation and hierarchical clustering analysis of metabolic data derived from four peanut testa with different color. (A) Pearson correlation coefficient R was applied in this project to evaluate reproducibility of biological replicate. (B) hierarchical clustering analysis of the abundance of 133 detected metabolites. Heatmap was drawn using the “pheatmap” package in the software R. Orange color indicates high abundance, green is low.

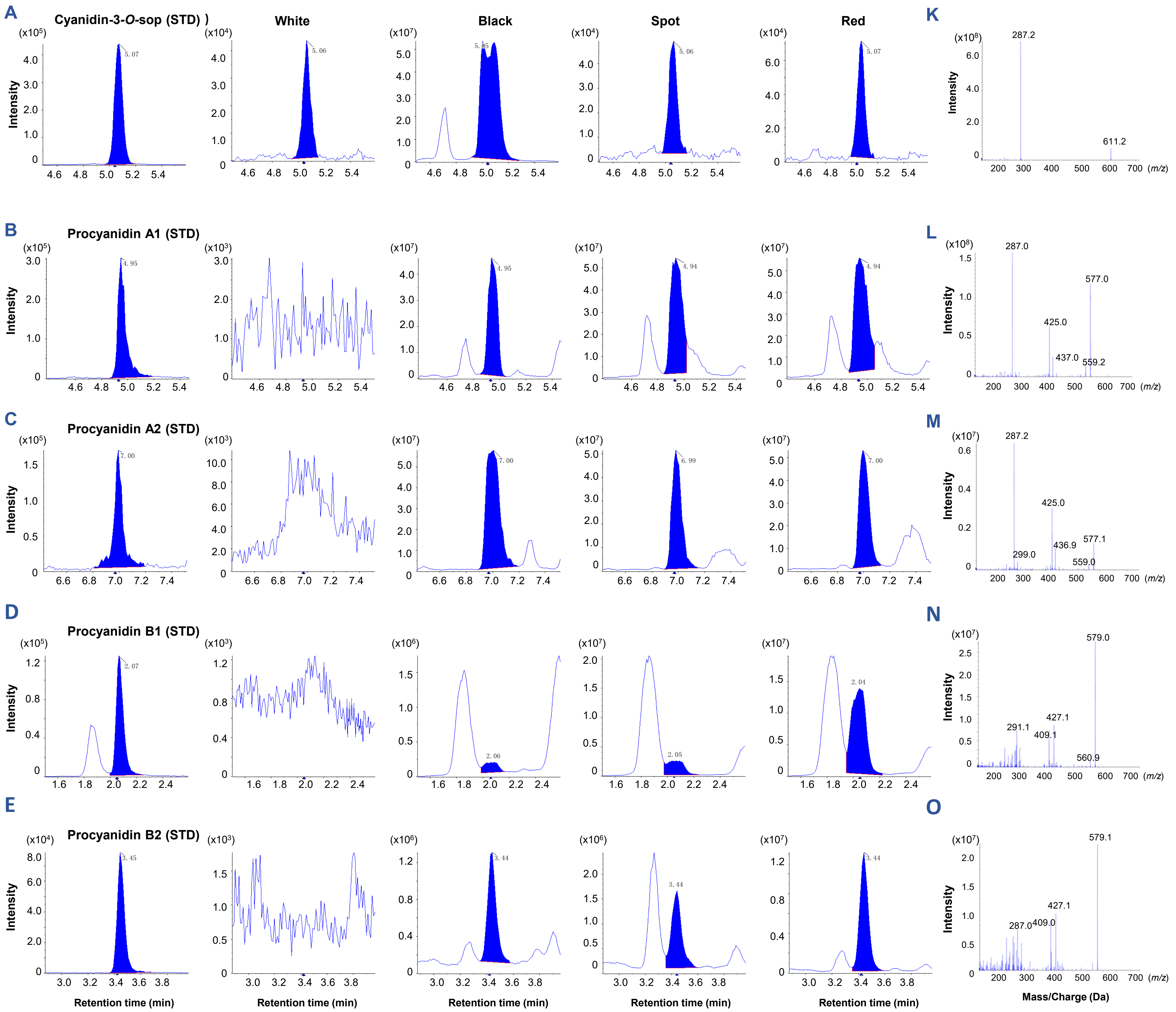

(to be Continued)

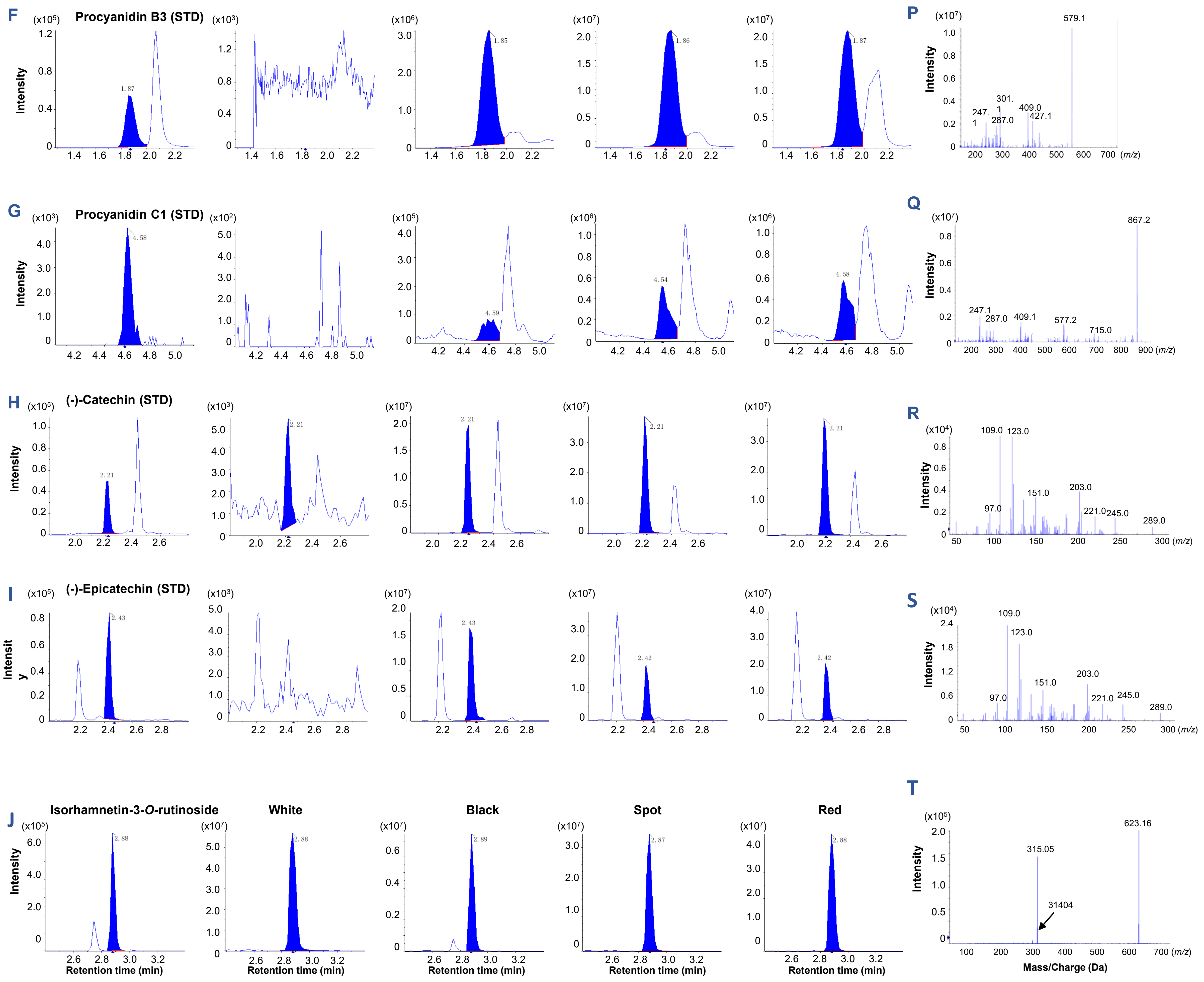

Figure S2. Extracted ion chromatography and mass spectrum of representative flavonoid metabolites in peanut seed coat. (A-J) Extracted ion chromatography of target metabolites in white, black, spot, and red peanut skins; (K-T) mass spectrum of standard compounds (STD).

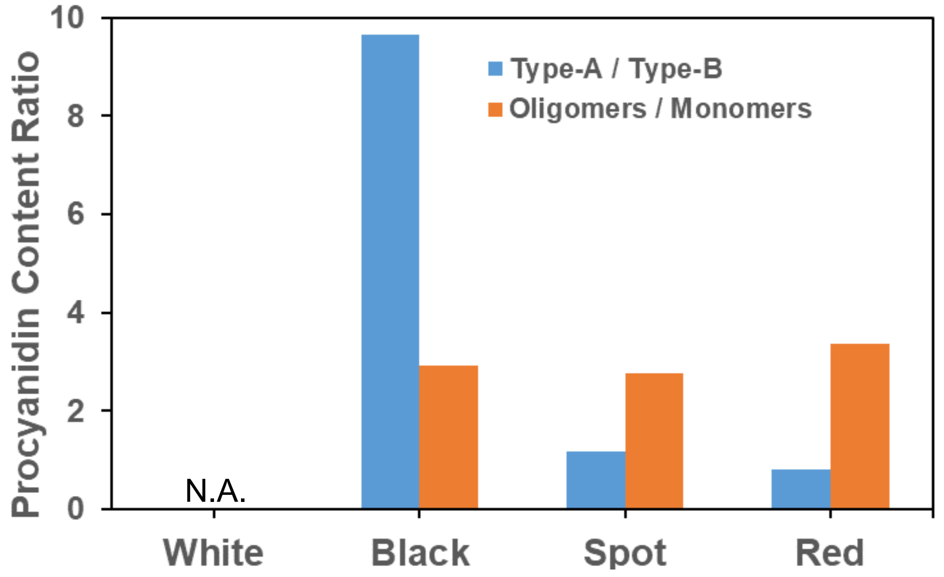

Figure S3. The content ratio of oligomeric procyanidins to monomeric flavan-3-ols in different peanut skins. In this study, procyanidins type-A refers to procyanidin A1 and procyanidin A2, while procyanidins type-B includes procyanidin B1, procyanidin B2, and procyanidin B3. The procyanidin monomers are (+)-catechin and (-)-epicatechin. The content ratio of Type-A to Type-B is calculated as " (total amount of the two procyanidin A)/(total amount of the three Procyanidin B). The content ratio of oligomers to monomers is calculated as "(sum content of the six procyanidins)/(sum content of (+)-catechin and (-)-epicatechin)". N.A. indicates the white testa sample data are not analyzed.

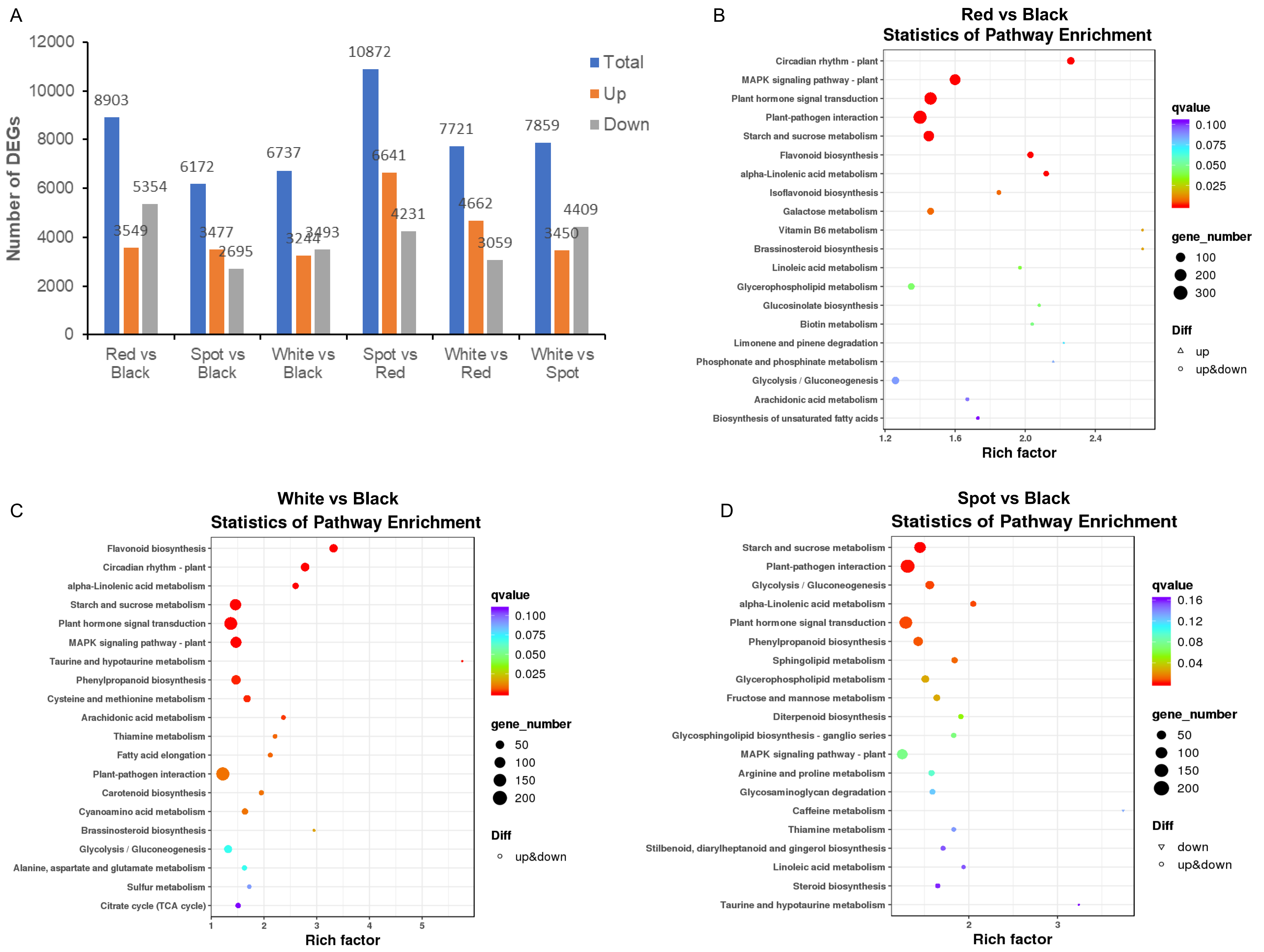

Figure S4. Analysis of differentially expressed genes (DEGs) between different peanut seed coat samples. (A)The number of DEGs between different samples. (B-D) KEGG pathway enrichment of DEGs in different comparison sets. Rich factor is calculated as “Rich factor=(Ratio of DEGs annotated to the term over all DEGs)/(Ratio of genes annotated to the term over all genes)" . A larger Rich factor indicates a more significant enrichment of the pathway. The color of the dots stands for q-value (adjusted p-value). The size of the dots represents the number of DEGs enriched in this pathway.

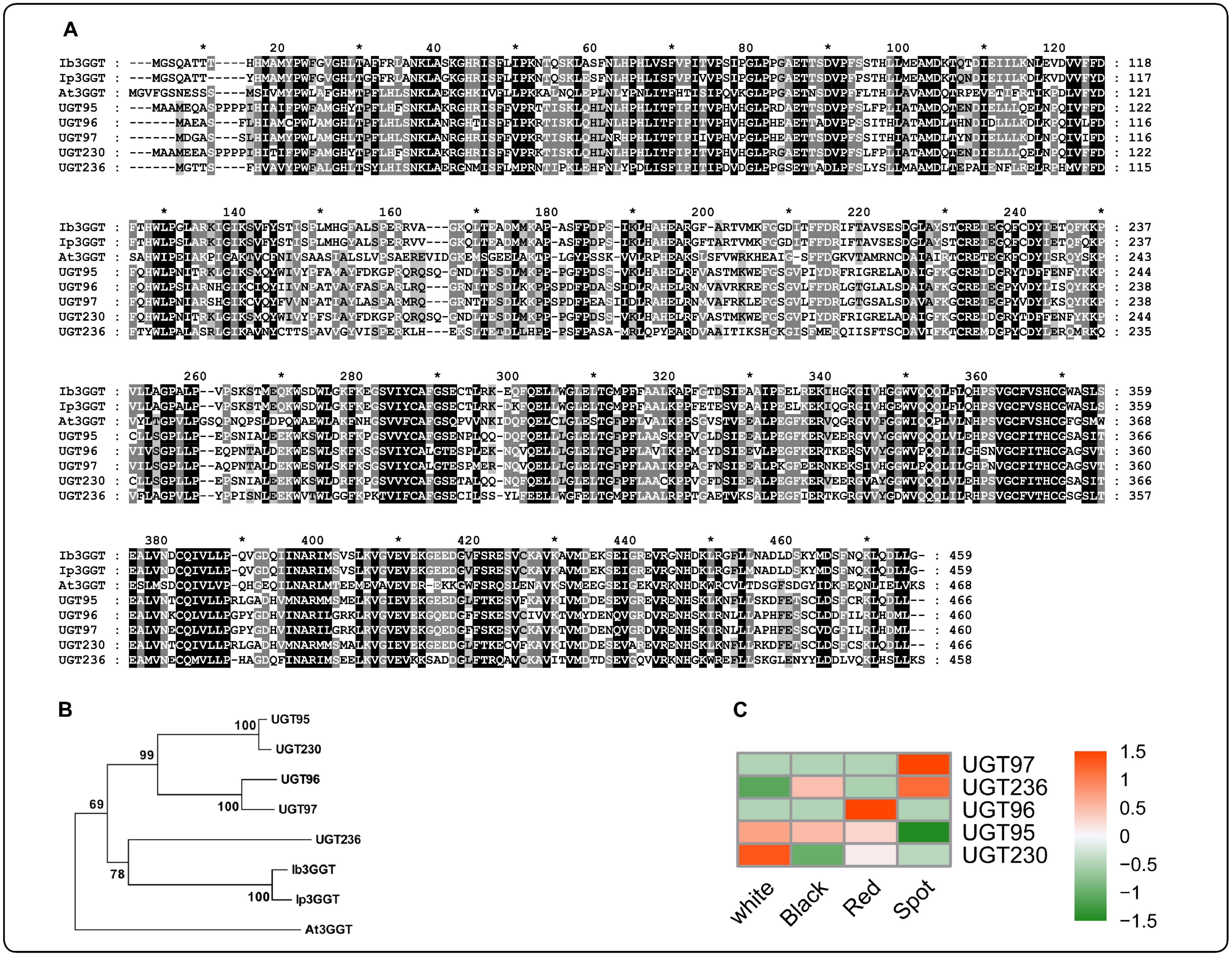

Figure S5. Analysis of candidate UDP-glycosyltransferases (UGTs) involved in peanut testa pigmentation. Multiple alignment (A) and phylogenetic analysis (B) of peanut UGTs with other functionally characterized plant UGTs. (C) Heatmap shows the expression level of candidate UGTs in different colored testa samples.
